# Impact of Cardiopulmonary Bypass Time on Postoperative Duration of Mechanical Ventilation in Patients Undergoing Cardiovascular Surgeries: A Systemic Review and Regression of Metadata

**DOI:** 10.7759/cureus.6088

**Published:** 2019-11-06

**Authors:** Rashid Nadeem, Shubham Agarwal, Shafaq Jawed, Ammar Yasser, Kamaleldin Altahmody

**Affiliations:** 1 Intensive Care Medicine, Dubai Hospital, Dubai, ARE; 2 Medicine, Chicago Medical School, North Chicago, USA; 3 Surgery, Jinnah Sindh Medical University, Karachi, PAK; 4 Medicine, Dubai Hospital, Dubai, ARE; 5 Cardiology, Tanta University, Tanta, EGY

**Keywords:** cardiovascular surgery, cardiopulmonary bypass time, aorta cross-clamp time, clinical outcome, mechanical ventilation

## Abstract

The objective of this study was to detect if cardiopulmonary bypass time duration has any impact on the duration of postoperative mechanical ventilation (MV). The study design was a systematic review and regression analysis of pooled data from previously published studies. All available data are from prospective, retrospective, cross-sectional, and observational studies. Participants included only patient/human studies. There were no interventions. PubMed and Cochrane libraries were searched by utilizing different combinations of keywords: cardiopulmonary bypass and mechanical ventilation. Inclusion criteria were: (1) English articles, (2) studies with an adult population that underwent cardiac surgeries using cardiopulmonary bypass (CPB), (3) studies where the duration of CPB is provided as well as the duration of mechanical ventilation. A regression analysis was performed on the metadata.

For the hours of MV, eight studies with 13 data sets (as some studies provide data in subgroups) were included for a total number of 989 subjects. The duration of CPB ranged from 55 to 173.5 minutes for these operations. Postoperative MV hours ranged from nine to 408 hours. Stepwise multiple regression analysis found that cardiopulmonary bypass time (CPBT), age, diabetes, male gender, and ejection fraction correlated with prolonged mechanical ventilation; CPBT was the most strongly correlated variable. Cardiopulmonary bypass time appears to affect clinical outcomes adversely and is associated with prolonged MV. Avoiding CPB or limiting it to a minimum may decrease the days of MV required.

## Introduction and background

Cardiopulmonary bypass (CPB) technology is used in many cardiovascular surgeries, including coronary artery bypass grafting (CABG) surgery [1]. Perioperative care of such patients is associated with substantial utilization of health care resources predominantly from prolonging the stay of patients in intensive care units and hospitals. These surgeries utilize CPB, which has been associated with some adverse effects. This is most likely due to exposure of blood to abnormal surfaces and conditions leading to systemic inflammatory responses [2]. More data are becoming available regarding these adverse effects. A majority of these surgeries are CABG, and, with the advent of the off-pump bypass, adverse effects can be avoided. However, data do suggest that off-pump surgeries may result in inferior graft patency and higher rates of repeated target-vessel revascularization [3-5].

Additionally, transcatheter valvular implantations are relatively newer techniques that do not require cardiopulmonary bypass technology. The clinical outcome of these complicated surgeries is dependent on multiple factors. Therefore, several studies are available addressing the impact of these factors on clinical outcomes, including CPB duration. We aim to pool data from these studies and perform a regression analysis to evaluate the effect of CPB duration on the need for prolonged mechanical ventilation (MV).

## Review

This analysis was performed and reported according to a prespecified protocol which was prepared in accordance with the preferred reporting items for systematic reviews and meta-analyses (PRISMA) statement. PubMed, Embase, Web of Science, and Cochrane Central Register of Controlled Trials databases were searched for studies published between January 1990 and August 2019, inclusively. The keywords were as follows: cardiopulmonary bypass time or CPBT and clinical outcomes, MV. An English language restriction was imposed on the search. Two reviewers screened citations and abstracts in duplicate and independently. The inclusion criteria encompassed adult population studies that have reported values for at least one of the outcomes of interest, and the study must have included data about the CPBT and reported the patient number for all groups. Case reports, case series, systematic reviews, and meta-analyses were excluded. Studies performed before 1990 were not included as CPB technology has changed significantly in the last three decades. A disagreement between the reviewers’ decisions regarding inclusion and exclusion were resolved through discussion. Two reviewers independently extracted variables from the identified studies, including publication details, country of origin, setting, study design, patient characteristics, interventions, methodological quality, compliance with the algorithm, and outcomes. CPBT duration was extracted from studies as a mean with standard deviation. For studies with data reported in median and interquartile range, mean and standard deviation were calculated utilizing methods outlined by Hozo et al. [6].

Some studies provided data in subgroups: patients with renal failure versus no renal failure or patients with atrial fibrillation versus patients without atrial fibrillation. Similarly, some studies provided data with a shorter duration of CPBT and longer duration of CPBT; therefore, two datasets were created for those studies. Study selection, data extraction, and statistical analysis were all done in accordance to previously published methodology for meta-analyses. For stepwise regression analysis of metadata, all studies' variable outcomes were selected as dependent variables, and CPBT and confounding variables were selected as independent variables, and IBM SPSS Statistics for Windows, version 26 (IBM Corp., Armonk, NY, USA) stepwise multiple regression analysis tool was utilized.

**Figure 1:**
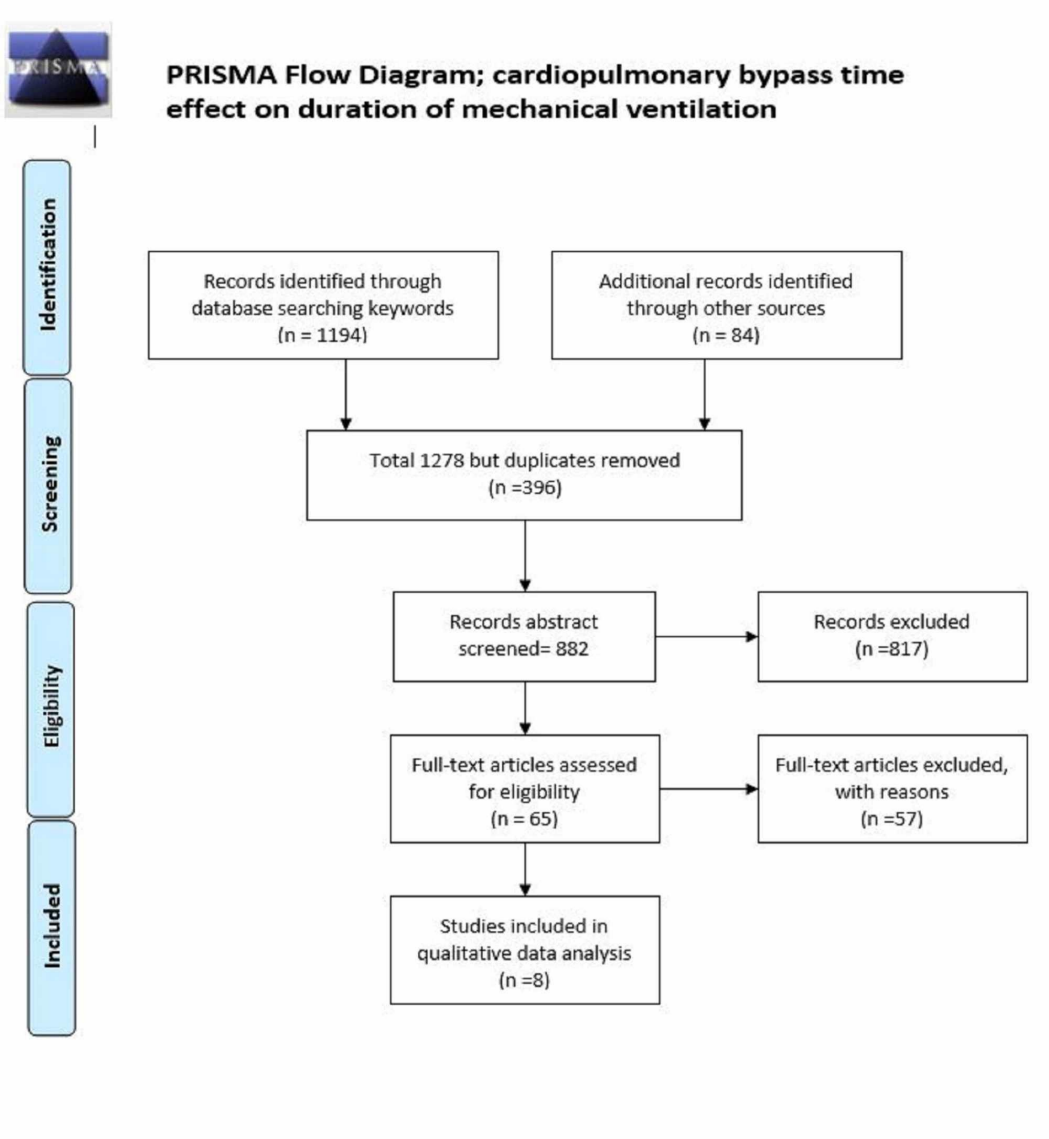
PRISMA flow diagram CPB time duration and MV duration Abbreviation: PRISMA, Preferred Reporting Items for Systematic Reviews and Meta-analyses.

For MV (hours), eight studies (Figure 1) with 13 data sets (as some studies provide data in subgroups) included a total number of subjects (N= 989) (Table 1). The duration of CPB ranged from 55-173.5 minutes for these operations. The incidence of postoperative MV hours ranged from nine to 408 hours. Stepwise multiple regression analysis found that CPBT, age, diabetes, male gender, and ejection fraction correlated with prolonged MV; CPBT was the most strongly correlated variable (Table 2).

**Table 1:** Studies with data about CPB and PMV Abbreviations: CPB, cardiopulmonary bypass; CPBT, cardiopulmonary bypass time; MECC, minimized extracorporeal circulation; MV, mechanical ventilation; PMV, postoperative mechanical ventilation.

Study name	Reason for grouping	Number of patients	CPBT mean	MV hours
Covino 2001 [7]		16	78	19.11
Erkut 2013 [8]	Beating heart	56	95	41
Erkut 2013 [8]	Non-beating heart	65	84	33
Malik 2006 [9]		25	103.4	15.84
Medved 2008 [10]		30	55	14
Møller 2010 [11]		163	64.6	9
Muñoz 2000 [12]	Survivors	142	88.5	48
Muñoz 2000 [12]	Survivors with complications	18	127	223
Muñoz 2000 [12]	Deaths	14	173.5	408
Remadi 2006 [13]	Conventional	200	65.4	9.7
Remadi 2006 [13]	Mini Jostra system	200	63.4	8.8
Schöttler 2007 [14]	Conventional	30	100.7	20.7
Schöttler 2007 [14]	MECC system	30	103.3	18
Total		989		

**Table 2:** Mechanical ventilation stepwise multiple regression analysis Abbreviations: AF, atrial fibrillation; CPBT, cardiopulmonary bypass time; CVA, cerebral vascular event; DM, diabetes mellitus; EF, ejection fraction; HTN, hypertension; MAP, mean airway pressure; MV, mechanical ventilation; SD, standard deviation.

Variable	Mean	SD	dataset (N)	Correlation	P value
MV	66.78	117.29	13	1	.
CPBT mean	92.44	31.78	13	0.88	.001
CVA	3.7		1		
Age	49.27	29.66	12	-0.772	.002
Men (%)	72.89	13.99	10	-0.671	.017
DM	19.9	3.44	7	0.746	.027
HTN	39.46	10.07	8	-0.389	.17
AF	28.16	15.78	4	-0.892	.054
Cross clamp	37.45	20.86	8	-0.463	.124
Temp	34.63	1.59	8	-0.332	.211
MAP	56.75	14.31	4	-0.9	.05
Blood flow	2.5	0	2		0
Euroscore	4.6	1.53	4	-0.866	.067
Score II	8.16	3.88	3	0.97	.079
EF	47.05	14.59	7	-0.837	0.009

Our results showed that a longer duration of CPB is associated with an increased incidence of a longer duration of MV. Similarly, multiple studies comparing on-pump versus off-pump cardiovascular surgery have documented adverse outcomes in on-pump conventional CPB patients [3,15-16]. 

Lastly, data suggest longer CPB leads to longer MV, and off-pump bypass is also associated with less time with MV after CABG [17,18].

Is there a cutoff limit for CPB duration above which MV duration is affected? Most studies, however, record data in terms of groups (less than 60 minutes or more than 120 minutes). Other studies presented data in terms of frequency number of patients with CPBT more than 120 minutes or 90 minutes and compare these outcomes. Therefore, it is unclear if less than 30 minutes have no adverse impact. Moreover, CPBT as a continuous variable was rarely used in these studies. Therefore, our analysis has a unique value that we have used CPBT as a continuous variable to estimate the extent of adverse impact on MV.

CPB has been shown to induce complement activation, endotoxin release, leukocyte activation, the expression of adhesion molecules, and the release of many inflammatory mediators, including oxygen-free radicals, arachidonic acid metabolites, cytokines, platelet-activating factor, nitric oxide, and endothelin [19]. This mechanism is postulated to translate into adverse clinical effects like prolonged MV.

We identified some weaknesses in our analysis. There was a paucity of studies with an expression of data in terms of two groups with shorter and longer time duration of CPB. This precludes us from performing a direct meta-analysis, although regression analysis does provide a relationship of time duration upon MV. Regression analysis on metadata without incorporation of each study population size makes the result independent of each study sample size and design. This may decrease the clinical strength of the results as larger sample size studies with better design have an equal impact as studies with smaller sample sizes. We should also mention that recent studies might have better clinical outcomes than earlier studies, as the quality and technology of oxygen membranes in CPB have been improving. We did not have enough studies with different types of CPB circuits. Therefore, we cannot perform a subgroup analysis. A similar effect from advancement in surgical techniques have another confounding effect. Despite this, our stepwise multiple regression provided an estimate of confounding variables: age, gender, and comorbid conditions, which provide better correlation. Moreover, there was no significant collinearity, making our results more robust. This highlights the importance of efforts to minimize the duration of CPB time or to consider off-pump surgeries if other risk profile also favors off-pump modality.

## Conclusions

CPB time duration appears to affect clinical outcomes adversely and is associated with prolonged MV. Increasing cytokine upregulation associated with longer exposure to CPB membranes may impact the respiratory system in a way similar to systemic inflammatory response syndrome. Therefore, avoiding CPB when feasible or limiting the duration to as little time as necessary when it is absolutely required may decrease the days of MV. The addition of newly developed filters to remove cytokines is an option in the near future.
